# Association between chronic bacterial airway infection and prognosis of bronchiolitis obliterans syndrome after hematopoietic cell transplantation

**DOI:** 10.1097/MD.0000000000013951

**Published:** 2019-01-04

**Authors:** Makiko Yomota, Noriyo Yanagawa, Fumikazu Sakai, Yuta Yamada, Noritaka Sekiya, Kazuteru Ohashi, Tatsuru Okamura

**Affiliations:** a Department of Respiratory Medicine, Tokyo Metropolitan Komagome Hospital, 3-18-22 Honkomagome, Bunkyo-ku, Tokyo, Japan; b Department of Radiology, Tokyo Metropolitan Komagome Hospital, 3-18-22 Honkomagome, Bunkyo-ku, Tokyo, Japan; c Department of Hematology, Tokyo Metropolitan Komagome Hospital, 3-18-22 Honkomagome, Bunkyo-ku, Tokyo, Japan; d Department of Infectious Disease, Tokyo Metropolitan Komagome Hospital, 3-18-22 Honkomagome, Bunkyo-ku, Tokyo, Japan.

**Keywords:** airway infection, bronchiolitis obliterans syndrome, hematopoietic stem cell transplantation

## Abstract

Bronchiolitis obliterans syndrome (BOS) is a rare pulmonary complication of hematopoietic stem cell transplantation (HSCT) with high mortality. Chronic bacterial airway infection (CAI) causes exacerbation and progression of several airway diseases, and bacterial airway colonization was shown to be associated with BOS after lung transplantation.

We assessed the association between CAI and clinical course in patients with BOS after HSCT. This retrospective study included 910 patients undergoing allogeneic HSCT between 2005 and 2013 at our institution. BOS diagnosis was reevaluated according to the 2014 US National Institutes of Health criteria. Sputum and bronchial lavage culture results, pulmonary function, and survival were compared between patients with and without CAI.

Median follow-up was 974.5 (261.5–2748.5) days. BOS was diagnosed in 27 (3.0%) patients, including 18 males. Median age at BOS diagnosis was 45 (40.5–58) years. Nine patients had ≥2 positive sputum cultures for bacteria or one positive bronchial lavage culture for nontuberculous mycobacteria (CAI+), whereas 9 patients had negative sputum/bronchial lavage culture or only one positive sputum culture (CAI−). Median change in forced expiratory volume in 1 s within 6 months after BOS diagnosis and overall survival were significantly worse in CAI+ patients than in CAI− patients (−250 vs +260 mL, *P* = .002, and 1340 days vs not reached, *P* = .04, respectively). No other factors including patient demographics or transplant protocol affected prognosis. There were no differences in clinical characteristics of patients with and without CAI, except for the time from transplantation to BOS diagnosis (214 vs 768 days for CAI+ and CAI−, respectively; *P* = .02).

CAI was associated with worse outcomes in patients with BOS after HSCT. Further prospective studies should assess the association between the airway microbiome and changes in pulmonary function after HSCT to improve prognosis.

## 1 Introduction

Bronchiolitis obliterans syndrome (BOS) is a small airway disease characterized by subepithelial inflammatory and fibrotic narrowing of the bronchioles. BOS is associated with various conditions such as autoimmune disorders, inflammatory bowel disease, drug side effects, inhalation of toxins, infections, post-lung transplantation, and post-hematopoietic stem cell transplantation (HSCT).^[1]^

BOS after HSCT was recognized in the 1980 s as the presence of irreversible fibrotic narrowing that selectively occurs in bronchioles.^[2,3]^ The clinical diagnostic criteria for BOS, which were first defined by the National Institutes of Health in 2005,^[4]^ was later modified in 2014.^[5]^ BOS after HSCT occurs rarely, with a prevalence of 2% to 7%;^[6]^ however, it has a poor prognosis: 5 year survival rate after the development of BOS was reported as 10%.^[7]^ There are currently no treatments established that could improve survival.

Several studies demonstrated that colonization with *Pseudomonas* species was associated with a high prevalence of BOS after lung transplantation.^[8]^ However, the influence of bacterial colonization on the clinical course of patients after the BOS diagnosis has not been well described.

An international clinical practice guideline for the diagnosis and management of BOS after lung transplantation, which was published in 2014, noted that patients with a pattern of early decline in pulmonary function might present with a more severe and aggressive BOS phenotype that is characterized by rapid progression and poor prognosis.^[9]^ We therefore hypothesized that bacterial colonization or chronic bacterial airway infection (CAI) might underlie this BOS phenotype, thereby contributing to the progression of the syndrome.

The aim of this study was to assess the efficacy of BOS management by evaluating the impact of bacterial airway infection on BOS progression by comparing the clinical course of BOS between patients with and without infection.

## 2 Materials and methods

### 2.1 Study population and data collection

This retrospective, observational study evaluated the medical records of patients who underwent HSCT between 2003 and 2015 at Tokyo Metropolitan Komagome Hospital after study approval was obtained from the ethics committee of the hospital (IRB No: 1785). The requirement of individual informed consent was waived because prior general consent for the use of medical information for research is obtained from all the patients who are referred to our hospital, while they are also provided with an option to opt out from the same. After HSCT, all patients underwent regular follow-up at least once a month. Those patients who were diagnosed with BOS by the treating clinician by May 2016 were eligible to be included in the study. The observational period for the study ended in May 2017.

Inclusion criteria were as follows:

1. diagnosis of BOS according to the criteria of the US National Institutes of Health revised in 2014;
2. available data on sputum specimen or bronchial lavage fluid in patients with no findings suggesting acute pulmonary infection; and
3. available thoracic computed tomography (CT) scans from before transplantation, before BOS diagnosis, and within 4 weeks after BOS diagnosis.

For diagnosis of BOS, inspiratory and expiratory high-resolution thoracic CT scans were captured. Exclusion criteria were as follows:

1. patients who developed severe systemic infection, hematological relapse, or multiple organ failure within 1 year after BOS diagnosis and
2. patients with other noninfectious pulmonary complications including idiopathic pneumonitis syndrome, drug-induced pneumonitis, and interstitial pneumonitis.

Diagnosis of BOS was reevaluated according to the criteria published by the US National Institutes of Health in 2014,^[5]^ which included the following criteria:

1. forced expiratory volume in 1 s (FEV_1_) / vital capacity ratio (FEV_1_%) < 0.7 or the fifth percentile of predicted
2. FEV_1_ < 75% of predicted values, with ≥10% decline in a span of less than 2 years. FEV_1_ should not correct to >75% of predicted with albuterol, and the absolute decline in corrected values should remain at ≥10% over 2 years
3. Absence of respiratory tract infection documented with evaluation by chest radiography, CT, or microbiologic cultures using specimens from sinus aspiration, upper respiratory tract viral screening, sputum, or broncho-alveolar lavage fluid, based on the specific clinical symptoms.
4. One of the 2 supporting features of BOS (a) evidence of air trapping on expiratory CT or small airway thickening or bronchiectasis by high-resolution chest CT or (b) evidence of air trapping by pulmonary function tests based on a residual volume (RV) >120% of the predicted values or an elevation in RV/total lung capacity over the 90% confidence interval. Patients who were diagnosed with BOS but did not meet the criteria were excluded from the study.

### 2.2 Radiological evaluation

Two radiologists and a pulmonologist reevaluated the CT scans that were obtained at least 3 times for each patient: before transplantation, at the time of BOS diagnosis, and after the diagnosis of BOS. Disagreements regarding the CT scans between the raters were resolved by consensus. The reevaluation included the appearance or progression of findings defined in the BOS diagnostic criteria including bronchiectasis, air trapping, and small airway wall thickening. Bronchiectasis was defined as a bronchial lumen that was wider than the diameter of the accompanying artery. Air trapping was diagnosed if the lung parenchyma remained lucent on the expiratory CT scan, exhibited less than the normal increase in attenuation after expiration, or was visualized as mosaic perfusion on the inspiratory scan. The diagnosis of small airway wall thickening was based on an increase in the thickness of the airway walls after the BOS diagnosis in comparison with the pretransplantation scan. Progression of CT findings was also evaluated by comparing CT at the time of BOS diagnosis with that after the BOS diagnosis.

### 2.3 Evaluation of infection

An accurate evaluation of bacterial colonization was not possible because of the retrospective study design. Therefore, symptomatic CAI, a cause of exacerbation and progression of airway diseases, was evaluated on the basis of the data of respiratory samples during the chart review of patients. At the time of assessment of respiratory samples, collected specimens were sent to the clinical laboratory and inoculated onto different media including trypticase soy agar with 5% sheep blood, GC II agar with hemoglobin and IsoVitaleX Enrichment). Other media were added for anaerobes, mycobacteria, and fungi based on clinical suspicion. Semi-quantitative methods were used for reporting the culture results.

CAI was defined on the basis of the European consensus definition for chronic *Pseudomonas aeruginosa* infection in cystic fibrosis, namely, at least 2 separate positive cultures of sputum or bronchial lavage fluid with at least a 1-month interval between the samples.^[10]^ For the diagnosis of nontuberculous mycobacteria (NTM), the American Thoracic Society's Official Statement of Diagnosis of Nontuberculous Mycobacterial Diseases was followed, in which one positive culture of bronchial lavage fluid was diagnostic.^[11]^ Positive cultures were diagnostic for CAI only when there were no symptoms suggestive of acute infection, such as high fever or new consolidation on chest X-ray.

### 2.4 Clinical variables and statistical analysis

Primary endpoints were death due to respiratory failure and decline in FEV_1_ after the diagnosis of BOS. The baseline data were extracted for all patients diagnosed with BOS to analyze risk factors for death by respiratory failure and to determine differences in patient characteristics according to the risk factors.

Analyzed background characteristics of patients were sex, age at the time of transplantation, smoking history, underlying hematologic diseases, stem cell source, type of donors, conditioning regimen, severity of acute graft-versus-host disease (GVHD), and presence of chronic GVHD in other organs. Characteristics of patients with BOS that were included in the analyses included the time of transplantation to the diagnosis of BOS, use of corticosteroids or other immunosuppressive agents at the time of diagnosis of BOS, level of serum immunoglobulin G, pulmonary function tests and chest CT findings at the time of BOS diagnosis, development of CAI, and treatment regimens for BOS. To evaluate outcomes after the diagnosis of BOS, the change in FEV_1_ within 12 months and survival time starting from transplantation until the time of death due to respiratory failure were evaluated.

All analyses were performed with JMP statistical computing package (SAS Institute, USA). The Shapiro–Wilk test was used to assess the normality of distribution of continuous variables. Means with standard deviation and medians with interquartile ranges were used to present normally and non-normally distributed variables, respectively. Numbers with percentages were used to present categorical data. Contingency tables were evaluated with Fisher exact test, and group mean values were analyzed with Wilcoxon rank-sum test. Survival between groups with and without CAI was compared by the log-rank test and the Cox proportional hazards model. Data were censored at the end of May 31, 2017. No patient was lost to follow-up, and those who were alive on May 31, 2017 were censored for overall survival. For overall survival analysis, death due to any cause other than respiratory failure was censored.

## 3 Results

### 3.1 Characteristics of patients with post-HSCT BOS

Between 2003 and 2015, 1032 patients underwent HSCT, including 910 and 122 patients receiving allogeneic and autologous transplantation, respectively. The baseline characteristics of patients who underwent allogeneic HSCT are presented in Table 1. Briefly, BOS was diagnosed in 27 (3.0%) of the 910 patients who underwent allogeneic HSCT and included 18 males and 9 females. There were no patients who underwent autologous HSCT and developed BOS. The median age at BOS diagnosis was 45 (40.5–58) years.

**Table 1:**
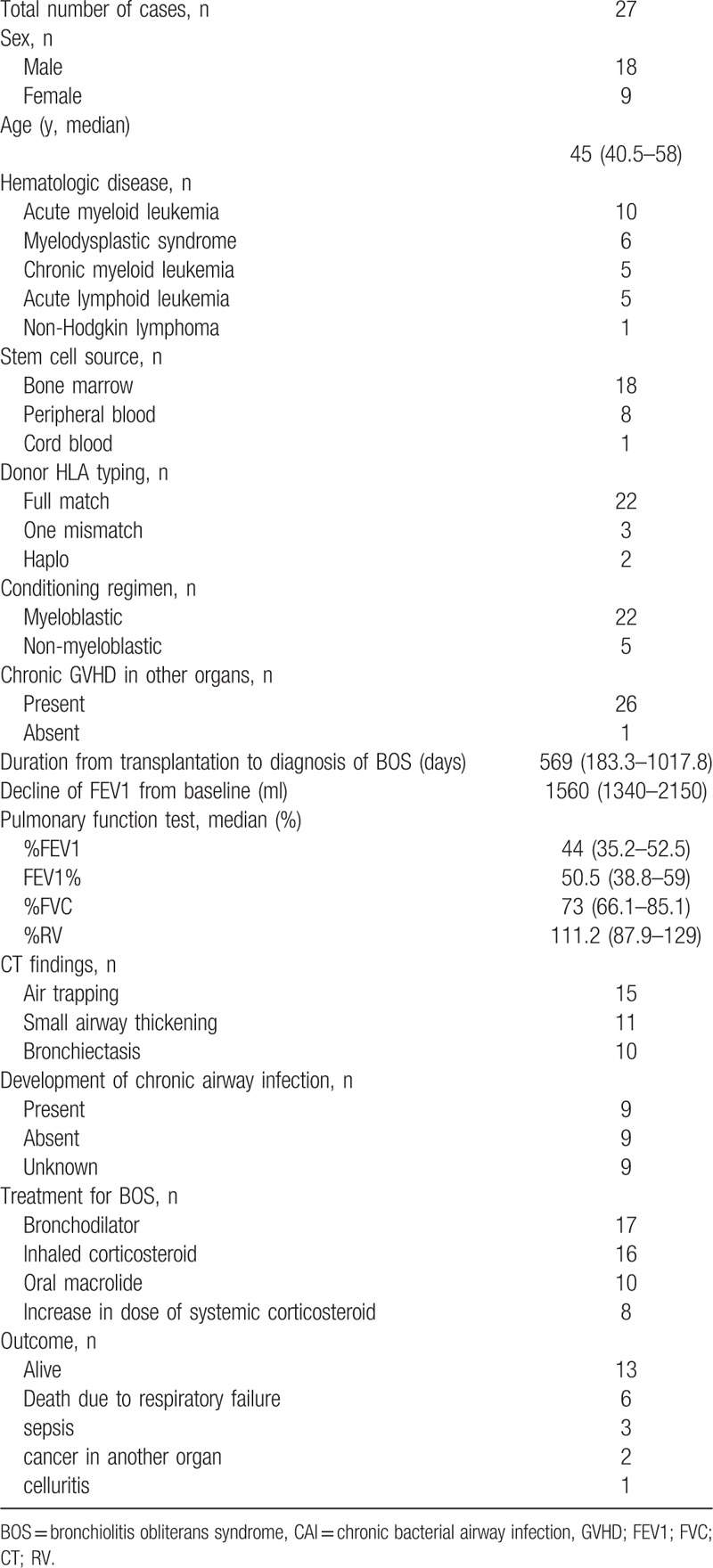
Background and baseline characteristics of patients with BOS.

The background and baseline characteristics of patients who were diagnosed with BOS are shown in Table 1. Briefly, the median duration from transplantation to the diagnosis of BOS was 569 (183.25–1017.75) days. At diagnosis, the median decline in FEV_1_ from baseline was 1560 (1340–2150) mL, and the median predicted %FEV_1_, FEV_1_%, and predicted %RV at the time of diagnosis were 41.4% (32.5–52.5%), 50.5% (38.8–59%), and 111.2% (87.9–129%), respectively.

According to the diagnostic criteria of BOS including the pulmonary function tests and the presence of another manifestation of chronic GVHD, there were no CT findings suggesting BOS at the time of BOS diagnosis in 6 patients. Among 21 patients who exhibited findings on CT suggestive of BOS, air trapping was the most common finding found in 15 patients, followed by small airway wall thickening and bronchiectasis in 11 and 10 patients, respectively.

For the treatment of BOS, bronchodilators, inhaled corticosteroids, and oral macrolides were administered in 17, 16, and 10 patients, respectively, and systemic corticosteroid dose was increased in 8 patients. Seventeen patients received more than 1 treatment specifically for BOS.

### 3.2 Characteristics of patients with CAI

**Figure 1:**
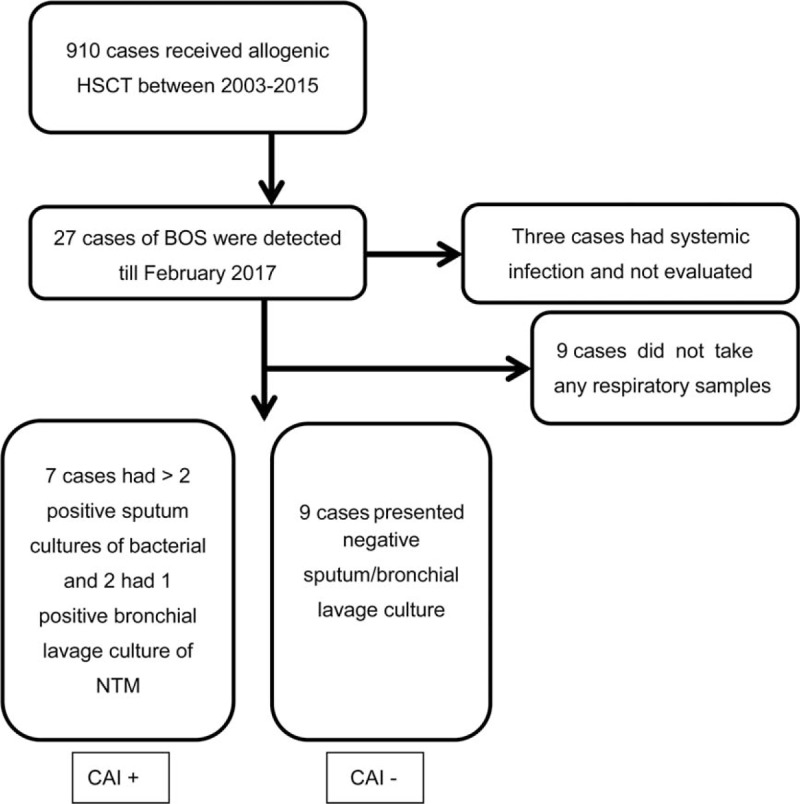
Flow diagram of the study for analysis of BOS patients with or without CAI. CAI = Chronic bacterial airway infection, HSCT = hematopoietic stem cell transplantation, NTM = nontuberculous mycobacteria.

As shown in Figure 1, among a total of 27 patients who received allogeneic HSCT and were diagnosed with BOS, 3 patients who developed systemic infections within 1 year after the diagnosis of BOS were excluded from the study. Among the remaining 24 patients, 9 patients had 2 or more positive sputum cultures for bacteria or one positive bronchial lavage culture for NTM (CAI+), and 9 patients had either negative sputum/bronchial lavage culture or only 1 positive sputum culture (CAI−), whereas respiratory samples could not be collected during the course of treatment in the remaining 6 patients, who were excluded from further analysis.

Pathogens causing CAI were detected in 9 patients (Table 2). Gram-negative rods or gram-positive cocci cultured from 7 patients were *Pseudomonas aeruginosa*, *Staphylococcus aureus*, *Haemophilus influenzae*, and *Stenotrophomonas maltophilia* in 3, 2, 1, and 1 patient, respectively. The other 2 patients had NTM including *Mycobacterium avium* and *Mycobacterium abscessus* in 1 patient each.

**Table 2:**
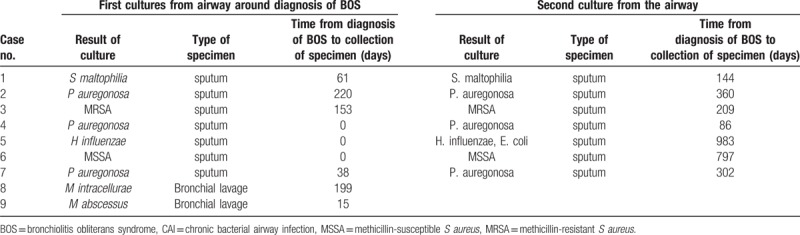
Culture results of sputum or bronchial lavage samples of patients with BOS and CAI.

### 3.3 Comparison of patients with and without CAI

The clinical presentation and outcomes of patients with or without CAI are shown in Tables 3 and 4. The median duration from the diagnosis of BOS to CAI was 209 (115–578.5) days. There were no significant differences in baseline clinical or transplantation characteristics at the time of diagnosis with BOS between the CAI+ and CAI− groups (Table 3). Additionally, there were no differences in the use of corticosteroids or other immunosuppressive agents, level of serum immunoglobulin G (IgG), or pulmonary function tests between the 2 groups. However, there was a significant difference in the time to BOS development from transplantation (ie, duration) between the CAI+ and CAI− groups (214 [46–553.5] and 768 [334–1166.5] days, respectively; *P* = .02; Table 4).

**Table 3:**
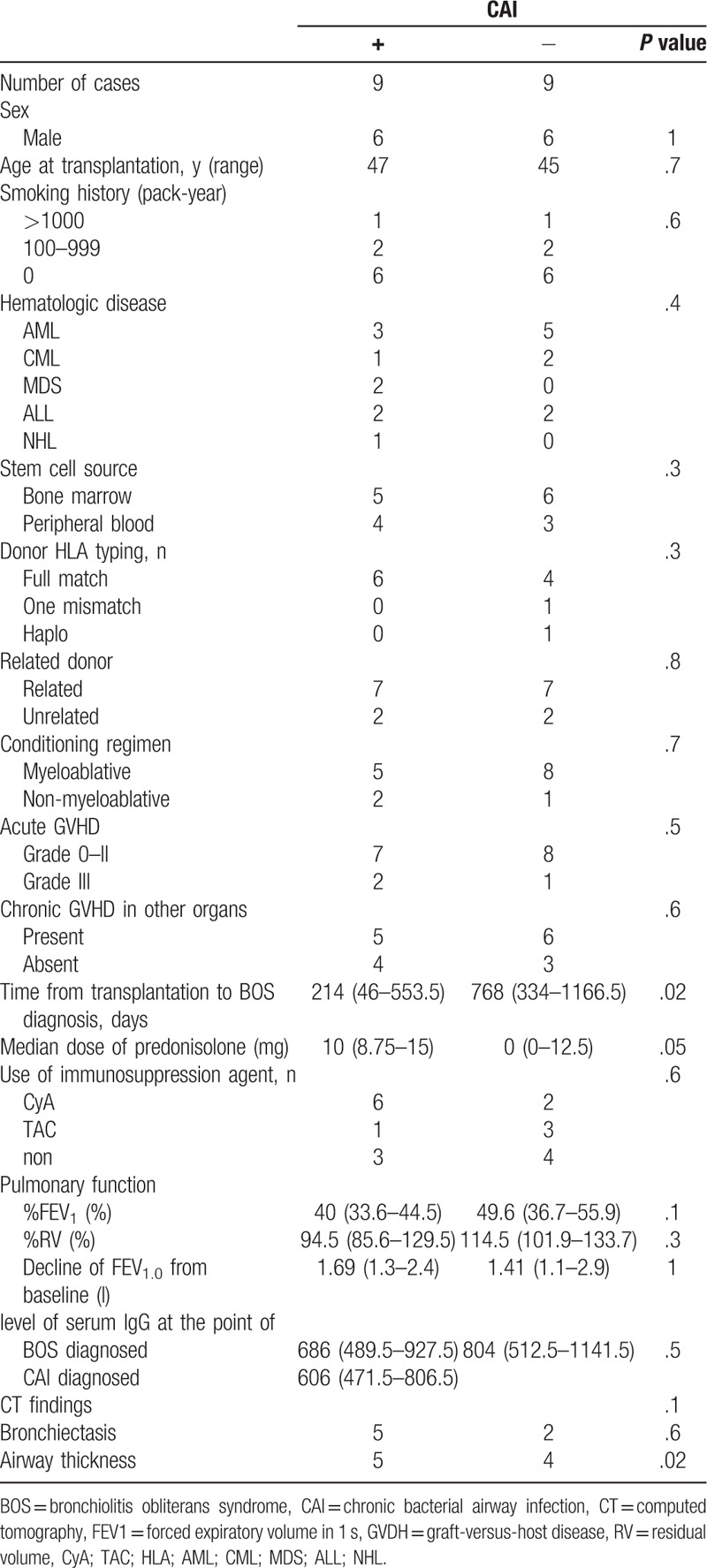
Comparison of background characteristics of patients with BOS with and without CAI.

**Table 4:**
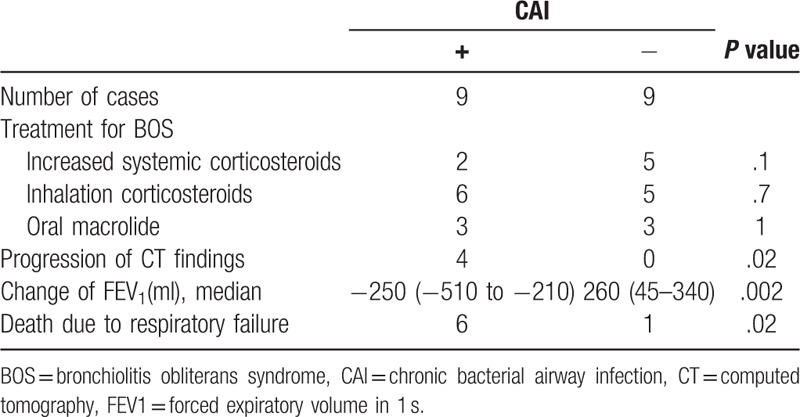
Clinical course and outcomes of BOS patients with and without CAI.

### 3.4 Clinical course after the diagnosis of BOS

**Figure 2:**
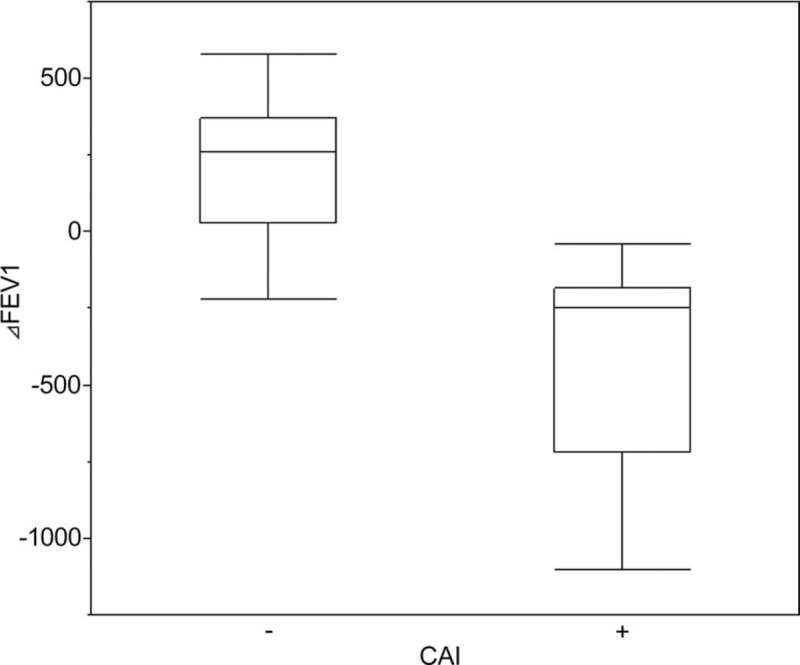
Kaplan–Meier analysis of deaths due to respiratory failure (solid line, CAI+; dotted line, CAI−). Symbols represent censored cases.

There were no statistical differences in treatment regimens for BOS (inhaled or systemic corticosteroids, bronchodilators, and macrolides) between the CAI+ and CAI− groups. The median time from the BOS diagnosis to the time of first sample collection in patients with CAI was 38 (0–176) days. Regarding pulmonary function tests, after the diagnosis and treatment of BOS, FEV_1_ was stable or slightly improved in the patients without CAI, whereas FEV_1_ in those with CAI decreased rapidly around the time a pathogen was detected. The median changes in FEV_1_ within 12 months after the diagnosis of BOS in patients with and without CAI were −250 (−720 to −185) and +260 (+30 to +370) mL, respectively (*P* = .002; Fig. 2). The progression of CT findings was observed in 4 of the 9 patients with CAI, whereas none of patients in the CAI− group exhibited signs of progression by CT (Table 4).

## 4 Survival

**Figure 3:**
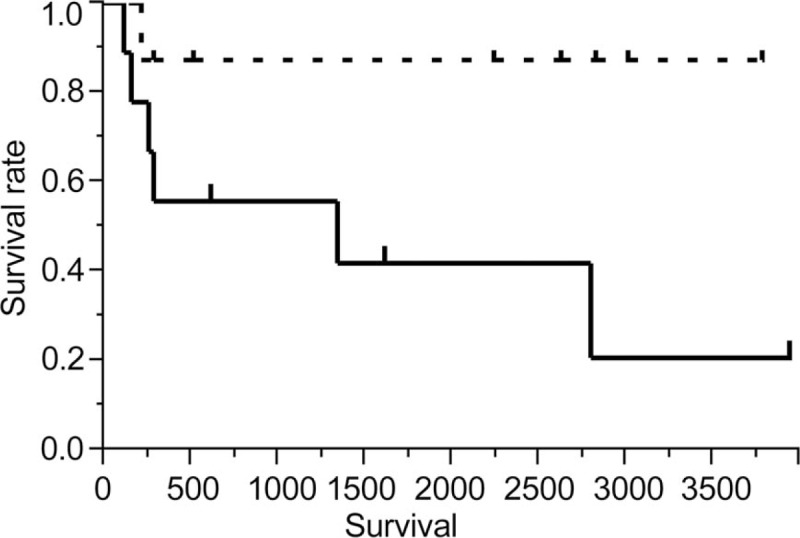
Box plot showing comparison of changes in FEV_1_ after diagnosis of BOS between patients with and without CAI. Notches on the box plots indicate 95% confidence interval of the median.

The median follow-up was 974.5 (261.5–2748.5) days. Among the 18 patients with BOS, 9 died during the observation period. The most common cause of death was respiratory failure that occurred in 7 patients. Causes of death for the other 2 patients were colorectal cancer and oral cancer at 580 and 3014 days after the diagnosis of BOS, respectively; both patients were censored from the survival analysis. Seven of the 9 patients with CAI died because of respiratory failure, whereas only 1 of the 9 patients without CAI died during the observation period. On the basis of the Kaplan–Meier analysis of deaths due to respiratory failure, overall survival was significantly worse in patients with CAI than in those without CAI (1340 days vs not reached, *P* = .04, Fig. 3). As shown in Table 5, by univariate Cox regression analysis, CAI was the only significant risk factor for death (hazard ratio 6.7, 95% confidence interval 1.1–127.5, *P* = .03). Other factors including male sex and FEV_1_ at the time of BOS diagnosis and the time from transplantation to the BOS diagnosis were not associated with survival. Although the time from transplantation to the BOS diagnosis was associated with the development CAI, CAI was an independent risk factor in multivariate analysis with CAI and the time from transplantation to the BOS diagnosis as covariables.

**Table 5:**
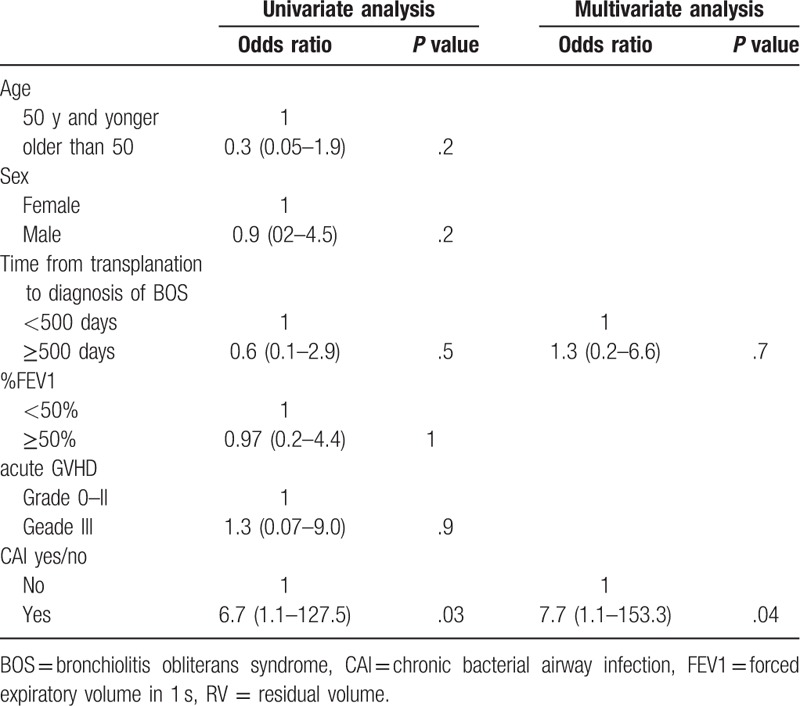
Cox regression analysis for survival of BOS patients.

## 5 Discussion

The current retrospective study revealed that CAI was associated with survival, FEV_1_ decline, and progression of CT findings in patients with BOS after HSCT. Conversely, there were no differences in the baseline characteristics, transplantation protocol, pulmonary function tests, and treatment regimens for GVHD and BOS between the patients with and without CAI.

CAI is common in patients with chronic respiratory diseases such as cystic fibrosis, bronchiectasis, and chronic obstructive pulmonary disease.^[12]^ Chronic bacterial colonization of the lower airways in patients with chronic obstructive pulmonary disease is well known to perpetuate inflammation and contribute to the disease progression. The interaction between bacterial infection, airway epithelial injury, and progression of airway disease was defined as the vicious circle hypothesis in 1992.^[13]^ Several studies showed that bacterial colonization or chronic bacterial infection increased the concentrations of inflammatory cytokines and led to the exacerbation and progression of chronic pulmonary and airway diseases.^[14,15]^

Our results are consistent with those of previous studies reporting adverse effects of bacterial airway colonization, including upregulation of cytokine expression in neutrophils and other inflammatory cells, resulting in persistent airway inflammation and fibrosis of small airways after lung transplantation.^[16,17]^ A retrospective study reported that airway colonization of *Pseudomonas* was a risk factor for the development of BOS after lung transplantation and that overall survival tended to be worse in the colonized patients although the *Pseudomonas* colonization was not a significant prognostic factor by itself.^[8]^ Another study reported that approximately half of patients with idiopathic pneumonitis syndrome after HSCT were reported to develop airway infection and that the survival of those patients was worse than patients without airway infections.^[18]^ In the current study, the development of CAI was not associated with level of serum immunoglobulin G, suggesting that the development and progression of BOS might not be correlated with systemic immune states, but also with changes in the airway biome and local immune response of the host's airway.

The current study has several limitations. First, pulmonary function tests after transplantation was scheduled at the discretion of the physician. Therefore, BOS diagnosis was missed in a number of patients, and no information was available on the presence of CAI or changes in pulmonary function before the development of BOS. Thus, whether there was an association between CAI and the development of BOS after HSCT remains unclear. Additionally, respiratory samples were not routinely evaluated; thus, we could not evaluate asymptomatic bacterial colonization of the airways or discuss the potential role of chronic respiratory viral and fungal infections. Second, the therapeutic interventions and the timing of BOS diagnosis were not based on a protocol. Because data regarding FAM therapy were not available at the time of treatment in some patients, BOS treatment was not based on a standardized protocol. Therefore, we could not evaluate potential advantages of specific treatment approaches. Finally, this was a retrospective study of a small number of patients, and the results cannot be generalized to other patients.

In the current study, the time from transplantation to the BOS diagnosis was shorter in patients with CAI than those without CAI. Previous studies reported that a short duration from transplantation to the development of BOS was a poor prognostic factor of BOS after HSCT.^[19,20]^ We did not find an association between the time from transplantation to the BOS diagnosis and survival, FEV_1_ decline, or progression of CT findings. Adjustment by multivariate analysis showed that the duration was not a confounder for the relationship between CAI and survival. However, because of the small number of patients, the multivariate adjustment may be vulnerable to false negative results for factors other than CAI. These findings implicate the presence of chronic occult infection before the diagnosis of BOS in the current study population, which might have led to BOS.

Macrolides are antibiotics with anti-inflammatory effects,^[21]^ and low-dose macrolide therapy was shown to dramatically improve survival in patients with diffuse pan-bronchiolitis.^[22]^ Several studies reported the efficacy of macrolides in improving lung function after lung transplantation in patients with BOS.^[23,24]^ Macrolides were also reported to have a prophylactic effect for BOS after lung transplantation in a cohort of 107 patients.^[25]^ Conversely, the efficacy of macrolide monotherapy in BOS after HSCT was investigated only in one clinical trial with a small number of patients,^[26]^ and a prophylactic effect for macrolides was not found in a randomized clinical trial that also revealed that macrolide therapy was associated with an increased risk of hematological relapse.^[27]^ A recent study reported that budesonide/formoterol therapy improved pulmonary function of BOS patients after HSCT,^[28]^ whereas other studies found that azithromycin in combination with inhaled fluticasone and montelukast reduced treatment failure and improved patient-reported outcomes of BOS after HSCT.^[29]^

This is the first study examining the association between bacterial airway infection and clinical course in BOS patients after HSCT, albeit the limitations of the small sample size and the retrospective study design. NTM infections can occur among patients with hematologic diseases,^[30]^ but the effect of NTM treatments in BOS patients was not reported previously. Whether there is a phenotype of BOS that develops in association with frequent airway infections or BOS is triggered by infection remains unclear. Prospective studies are necessary to further analyze the association between the airway biome and changes in pulmonary function after HSCT with the aim of improving prognosis in patients after HSCT.

In conclusion, bacterial airway infection was associated with progression and poor outcomes in patients with BOS after HSCT. Routine detection and management of airway biome after HSCT should be considered as a strategy for the treatment as well as the prevention of BOS.

## Acknowledgments

We thank Drs Yuho Najima, Noriko Doki, Takeshi Kobayashi, and Kazuhiko Kakihana for their roles as attending physicians of the study patients. We also acknowledge Makoto Saito for his valuable feedback on the statistical analyses.

Dr Yomota reports personal fees from Chugai Pharmaceutical Co., Ltd and ONO Pharmaceutical Co., Ltd from null, outside the submitted work.

## Author contributions

**Conceptualization:** Makiko Yomota, Noriyo Yanagawa, Fumikazu Sakai.

**Data curation:** Makiko Yomota, Noriyo Yanagawa, Fumikazu Sakai, Yuta Yamada, Kazuteru Ohashi.

**Formal analysis:** Makiko Yomota, Noriyo Yanagawa.

**Investigation:** Makiko Yomota.

**Methodology:** Makiko Yomota, Fumikazu Sakai, Noritaka Sekiya.

**Project administration:** Makiko Yomota.

**Resources:** Makiko Yomota.

**Software:** Makiko Yomota.

**Supervision:** Noriyo Yanagawa, Fumikazu Sakai, Kazuteru Ohashi, Tatsuru Okamura.

**Validation:** Noriyo Yanagawa, Fumikazu Sakai, Noritaka Sekiya, Kazuteru Ohashi.

**Visualization:** Makiko Yomota.

**Writing – original draft:** Makiko Yomota.

**Writing – review & editing:** Makiko Yomota.

Makiko Yomota orcid: 0000-0002-1616-9893.
